# Expression profiling of the phenylalanine ammonia-lyase (*PAL*) gene family in *ginkgo biloba* L.

**DOI:** 10.1080/15592324.2023.2271807

**Published:** 2023-10-30

**Authors:** Xiaoge Gao, Yaping Hu, ZhiBiao Xu, Daqing Peng, Qirong Guo

**Affiliations:** a Co-Innovation Center for Sustainable Forestry in Southern China, Nanjing Forestry University, Nanjing, China; b Yancheng forest farm, Yancheng, Jiangsu, China

**Keywords:** Evolution, flavonoids, ginkgo, gene expression, phenylalanine ammonia-lyase

## Abstract

The *PAL* gene family plays an important role in plant growth, development, and response to abiotic stresses and has been identified in a variety of plants. However, a systematic characterization is still lacking in *Ginkgo biloba*. Using a bioinformatics approach, 11 *GbPAL* members of the *PAL* gene family identified in ginkgo were identified in this study. The protein structure and physicochemical properties indicated that the *GbPAL* genes were highly similar. Based on their exon-intron structures, they can be classified into three groups. A total of 62 cis-elements for hormone, light, and abiotic stress responses were identified in the promoters of *GbPAL* genes, indicating that *PAL* is a multifunctional gene family. *GbPAL* genes were specifically expressed in different tissues and ploidy of ginkgo. These results provide a theoretical basis for further studies on the functional expression of the *GbPAL* genes.

## 1. Introduction

Phenylalanine Ammonia-Lyase (PAL, EC 4.3.1.24) is a critical enzyme in the secondary metabolism of plants that plays a crucial role in their stress resistance.^1^ *PAL*, which is one of the earliest enzymes in the phenylpropanoid biosynthesis pathway, is responsible for regulating the synthesis of various secondary metabolites such as flavonoids, lignin, and phenolic compounds.^2–4^ Since its initial extraction from *Hordeum vulgare* in 1961, *PAL* has been successfully isolated and purified from higher plants, including *Solanum tuberosum* and *Nicotiana tabacum*.^5–7^ A significant positive correlation between *PAL* activity and flavonoid content was reported in *Malus pumila* fruits in 1996.^8^ In *Arabidopsis thaliana*, *AtPAL1* and *AtPAL2* are highly expressed in roots and flowers, and their expression levels are influenced by nitrogen stress and temperature changes, which lead to the accumulation of flavonoids.^9^

The deciphering of more plant genomes has provided a scientific foundation for exploring the *PAL* gene family. The *PAL* gene family has been extensively studied in several plants, such as rice,^10^ *Medicago sativa*,^11^ *Vitis vinifera*,^12^ and *Cuminum cyminum*.^13^ However, the number of *PAL* gene family members varies significantly among different plants, with higher gene copy numbers in potatoes and lower gene copy numbers in *A. thaliana*, *Oryza sativa*, *V. vinifera*, and *N. tabacum*.^7,14,15^ There are four genes in the *PAL* gene family in the model plant *A. thaliana*: *PAL1* (AT2G37040), *PAL2* (AT3G53260), *PAL3* (AT5G04230), and *PAL4* (AT3G10340).^16^ *AtPAL1*, *AtPAL2* and *AtPAL4* play important roles in lignin synthesis in the plant vascular system, and *AtPAL1* and *AtPAL2* play important roles in flavonoid biosynthesis.^9^

*Ginkgo biloba* L., also known as a “living fossil”, is the sole representative of the ginkgo family and a typical example of gymnosperms with a vast and intricate genome.^17,18^ Ginkgo is widely used as an ornamental tree and a medicinal plant. Flavonoids are the primary economic components of *Ginkgo biloba* extract (GBE) and are extensively employed in the pharmaceutical industry, health products, food additives, functional beverages, and cosmetics.^19^ However, despite the discovery of a highly precise ginkgo reference genome, the *PAL* gene family in ginkgo has not been systematically explored. Investigating the *PAL* gene family of ginkgo can help unravel the disparities in the *PAL* gene family between gymnosperms and angiosperms.

The objective of this investigation is to identify the members of the *PAL* gene family in the ginkgo genome, and conduct a bioinformatics analysis of their phylogeny, gene structure, conserved motifs, and cis-acting elements utilizing the latest ginkgo genome-wide search approach. Furthermore, based on our previously obtained haploid transcriptome data, we compared the expression of each *GbPALs* with the diploid ginkgo genome and performed expression analysis for different sites and under different environmental stresses, respectively. Finally, we utilized quantitative reverse transcription-polymerase chain reaction (qRT-PCR) to assess the expression specificity of *GbPALs* in different tissues, and analyze the variations in expression levels among different ploidy levels of the ginkgo genome.

## 2. Materials and methods

### 2.1. Identification of PAL genes in ginkgo

First, the protein data of the whole-genome sequence of ginkgo were downloaded from NGDC (https://ngdc.cncb.ac.cn/). *AtPALs* sequences were used as reference sequence for ginkgo genome sequencing, using tools available on the NCBI website. The *PAL* gene and protein sequence of *A. thaliana* were downloaded from the TAIR12.0 website. Initial candidate genes that may contain *PAL* structural domains in ginkgo were identified through comparisons. Candidate *GbPALs* containing *PAL* structures were then identified using HMMER 3.0, by selecting default parameters (E-value threshold: 0.01; Alignment mode: local alignment; Search mode: heuristic search.) for screening from the Pfam database. All candidate *GbPAL* protein sequences were further examined using the Simple Molecular Architecture Research Tool (SMART, http://smart.embl-heidelberg.de/) and the Conserved Domains Database (CDD). The sequences of candidate *PAL* genes retrieved with missing the Lyase_aromatic domains (PF00221) were discarded to identify the members of the *GbPAL* gene family. The ExPASy (https://web.expasy.org/protparam/) server was used to calculate the molecular weight (MW) and isoelectric point (pI) of the proteins. The ProComp 9.0 database was used to predict the subcellular localization of PAL proteins. A per-residue confidence score (pLDDT) of 0–100 was generated using AlphaFold (https://alphafold.com/) to predict the tertiary structure of GbPAL proteins.

### 2.2. *Phylogenetic analysis of* GbPAL *genes*

PAL proteins from *A. thaliana*, *N. tabacum*, *Zea mays*, *Oryza sativa*, *Populus trichocarpa*, *V. vinifera*, *Juglans Regia*, *Picea abies* and *ginkgo* (Protein sequences in Supplementary Data Table S1) were used to construct phylogenetic trees. The 73 protein sequences were aligned using the Clustal W function of the MEGA 11 software, with default parameters selected (Scoring matrix: BLOSUM62; Gap opening penalty: 15; Gap extension penalty: 6.66; Delay divergent cutoff: 30%; Residue-Specific penalty: OFF; Hydrophilic penalty: OFF; DNA transition weight: 0.5; Use negative matrix: OFF; Maximum iterations: 1000). PAL protein sequences from nine different species were constructed using the neighbor joining (NJ) method, and 1000 bootstrap replicates were performed. The phylogenetic tree was visualized using the Evolview website (https://www.evolgenius.info/evolview/).

### 2.3. Gene structure and Conserved MotifAnalysis

MEME (http://meme-suite.org/tools/meme) was used to search *GbPAL* motifs; the number of searches was 20 and all other parameters were set to the same default settings. The sequences used in this study appeared at least once. Conserved patterns and gene structures were mapped using the TBtools software.

### 2.4. Analysis of the cis‑regulatory elements in the promoter

The cis-acting elements were analyzed in the 2000 bp sequence upstream of the candidate *PAL* gene start codon using the PlantCARE (http://bioinforma ics.psb.ugent.be/webtools/plantcare/html/) software. The TBtools software was used to determine the distribution of cis-elements and to draw the pattern.

### 2.5. Ginkgo PAL transcriptome analysis

Ginkgo root, stem, leaf, flower, seed, haploid and diploid transcriptome data was obtained from the NCBI by through decomposition of SRX4132865 for roots, stems, and leaves:^20^ SRR2147720,^21^ GSE128653,^22^ SRP337737^23^ for flowers, seeds, and haploid and diploid ginkgo. TPM values were calculated using Kallisto v0.44.0 (California, USA)^24^ and homogenized using Log2 (TPM +1) for different transcriptome data, and expression heat maps were plotted using TBtools.^25^

### 2.6. qRT-PCR for GbPAL

In April 2022, the flowers of five male ginkgo plants were collected from the Nanjing Forestry University campus and the root, stem, leaf and seed material of five female ginkgo plants were collected in August. Nanjing Forestry University (118.481 E, 32.444 N) is located in Nanjing, Jiangsu Province, China. The annual average temperature is 15.7°C, and the annual accumulated temperature (≥10°C) is 4897°C. The annual average precipitation is 971.7 mm, with spring and summer accounting for more than 70%, and autumn and winter accounting for only 30%. The annual frost-free period is 233d. Three biological replicates of each tissue sample were rapidly frozen in liquid nitrogen for total RNA extraction. Total RNA was extracted using the TRIzol reagent (Invitrogen Scientific, Inc., Carlsbad, CA, USA), according to the manufacturer’s protocol. RNA quality was assessed using an Agilent 2,100 Bioanalyzer (Agilent Technologies, Palo Alto, CA, USA) and RNase-free agarose gel electrophoresis. The extracted ginkgo RNA was reverse-transcribed using the PrimeScript^TM^ 1st Strand cDNA Synthesis Kit (TaKaPa, Dalian) and stored in a −20°C refrigerator. *GbCHS* (gene ID: AY496931.1) served as the endogenous control. According to the CT value, the relative expression of all *GbPAL* genes under different conditions was calculated using the 2^−ΔΔCt^ method and visualized using TBtools.

Based on the gene structure of *GbPAL*, qRT-PCR primers (Primer sequences in Supplementary Data Table S2) were designed using NCBI-blast, qRT-PCR was performed using SYBR Green Master Mix (Applied Biosystems), and samples were run in a CFX96 Real Time System (Applied Biosystems). The cycling conditions were as follows: denaturation at 95°C for 5 s, annealing at 55°C for 15 s, and extension at 65°C for 5 s. To ensure the accuracy of the results, three biological replicates and three technical replicates were used for each sample. The PCR products from each sample were verified through DNA sequencing.

### 2.7. Protein-protein interaction networks analysis

Utilizing the string website(https://cn.string-db.org/), we downloaded protein interaction relationship of *A. thaliana* and use it reference. Obtained data into Cytoscape software(v3.1.0)^26^ for visualization.

## 3. Results

### 3.1. Identification of GbPAL genes

We conducted a BLAST search of the ginkgo genome using *PAL* sequences from *A. thaliana* to identify *PAL* genes in ginkgo. Thirteen putative *PAL* genes were initially identified and subsequently confirmed to possess the *PAL* domain through submission to CDD, Pfam, and SMART (Supplementary Figure S1). Ultimately, we identified 11 members of the *GbPAL* gene family, which were designated as *GbPAL1-GbPAL11* in accordance with their gene ID (Table 1). The coding sequence length of the *GbPAL* genes ranged from 1650 bp (*GbPAL8*) to 2505 bp (*GbPAL3*), with an average of 699 amino acids (AA) per PAL protein. All PAL proteins had an isoelectric point (pI) less than 7 and were mildly acidic. While all *PAL* proteins except for *GbPAL5* and *GbPAL4* were stable (Instability Index < 40), their grand average of hydropathicity (GRAVY) ranged from −0.31 (*GbPAL4*) to 0.021 (*GbPAL11*), indicating that 81.8% (9/11) of *PAL* proteins were hydrophilic (with negative values for hydrophobic proteins). As no signal peptide was detected, the ginkgo *PAL* proteins were all intracellular proteins. Furthermore, the subcellular localization prediction indicated that all *PAL* proteins were located in the cytoplasm, except for *GbPAL10*, which was localized in the nucleus.

**Table 1.** The statistics of the ginkgo *PAL* gene family.

Gene name	Gene id	CDS (bp)	AA	pI	Instability Index	GRAVY	Signal Peptide	Subcellular Localization
*GbPAL1*	evm.model.chr6.121	2181	727	6.06	30.71	−0.255	No	Cytoplasm
*GbPAL2*	evm.model.chr8.697	2199	733	5.99	32.47	−0.171	No	Cytoplasm
*GbPAL3*	evm.model.chr8.2163	2505	835	6.21	31.96	−0.211	No	Cytoplasm
*GbPAL4*	evm.model.chr9.1603	2379	793	6.09	47.28	−0.31	No	Cytoplasm
*GbPAL5*	evm.model.chr9.1620	2379	793	6.29	47.71	−0.307	No	Cytoplasm
*GbPAL6*	evm.model.chr10.554	2196	732	5.96	32.37	−0.245	No	Cytoplasm
*GbPAL7*	evm.model.chr10.571	1794	598	6.31	31.54	−0.09	No	Cytoplasm
*GbPAL8*	evm.model.chr10.584	1650	550	6.03	31.89	−0.112	No	Cytoplasm
*GbPAL9*	evm.model.chr11.1054	2202	734	6.1	28.99	−0.187	No	Cytoplasm
*GbPAL10*	evm.model.chr12.1405	1758	586	6.52	38.01	0.012	No	Nucleus
*GbPAL11*	evm.model.chr12.1408	2073	691	6.06	38.11	0.021	No	Cytoplasm

**Figure 1.**
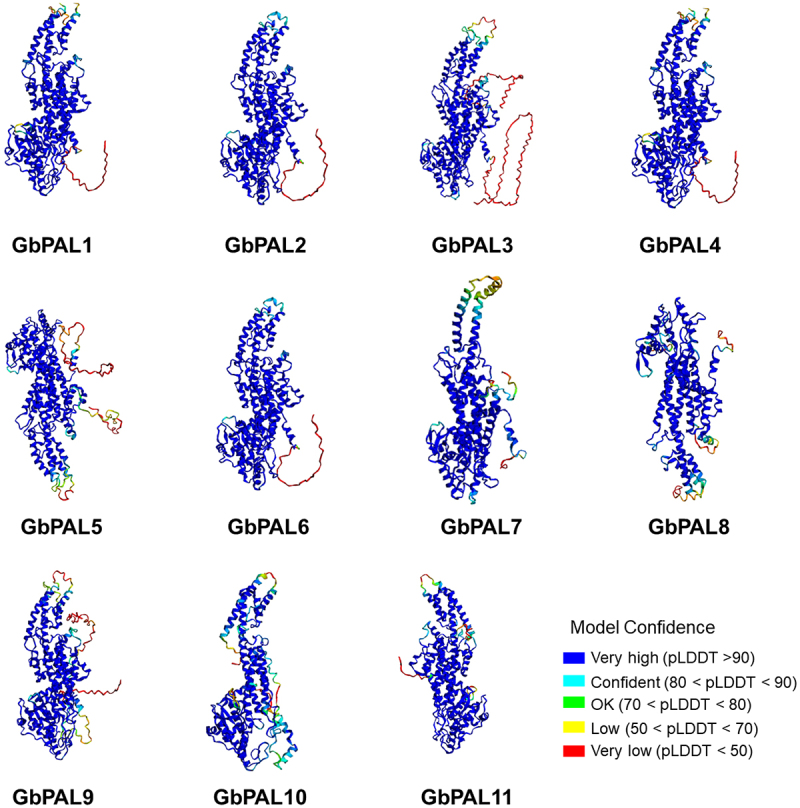
The tertiary structure of GbPAL proteins. The blue to green color indicates the confidence level from high to low, respectively.

A per-residue confidence score (pLDDT) of 0–100 was generated using AlphaFold to predict the tertiary structure of the GbPAL proteins (Figure 1). The tertiary structures of the 11 GbPAL proteins were highly similar.

### 3.2. Phylogenetic analysis of the GbPAL gene family

**Figure 2.**
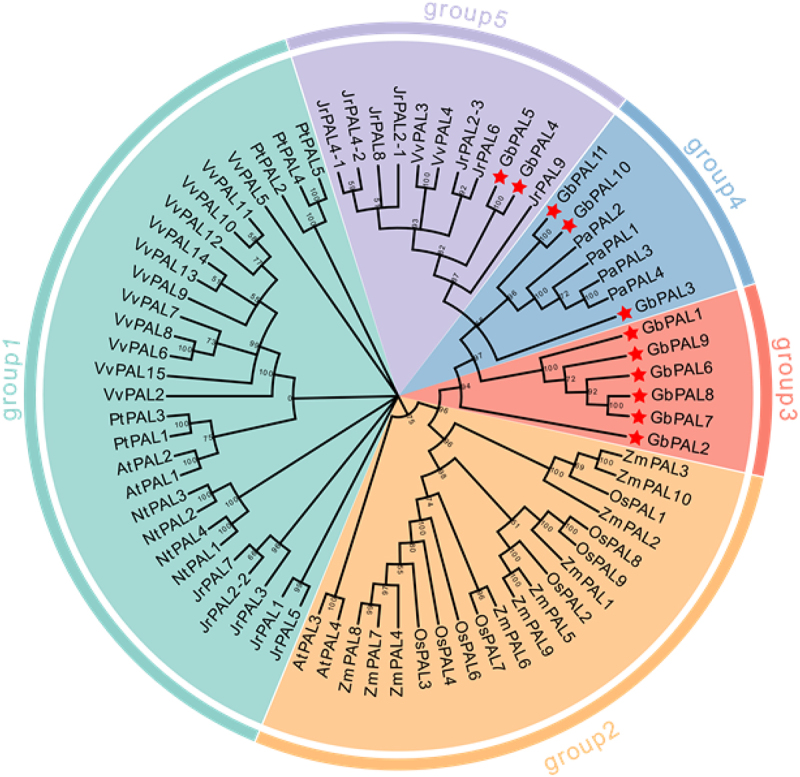
Phylogeny of the *PAL* gene family in different species. The conserved PAL proteins from *Arabidopsis thaliana*(At), *nicotiana tabacum*(Nt), *Zea mays*(Zm), *Oryza sativa*(Os), *populus trichocarpa*(Pt), *Vitis vinifera*(Vv), *Juglans Regia*(Jr), *Picea abies*(Pa), and *ginkgo biloba*(Gb).

To elucidate the phylogenetic relationships among *PAL* proteins from various species, we employed 73 PAL protein sequences to construct a phylogenetic tree using the neighbor-joining (NJ) method with 1000 bootstrap replications. The sequences were obtained from a diverse set of organisms, including *A. thaliana*, *N. tabacum*, *O. sativa*, *P. abies*, *Z. mays*, *P. trichocarpa*, *V. vinifera*, *J. Regia*, and ginkgo (Figure 2).

Based on an exhaustive comparison of the nucleotide sequences, the *PAL* genes were classified into five distinct groups. Notably, *GbPAL2*, *GbPAL6*, *GbPAL9*, *GbPAL1*, *GbPAL8*, and *GbPAL7* from ginkgo were found to cluster together in group 3, forming a separate category. In addition, *GbPAL3*, *GbPAL11*, and *GbPAL10* were observed to cluster with *PaPAL*, indicating a close relationship between these genes. This relationship was further substantiated by the fact that both ginkgo and *P. abies* are gymnosperms.

### 3.3. Analysis of conserved motifs and gene structures of GbPALs

**Figure 3.**
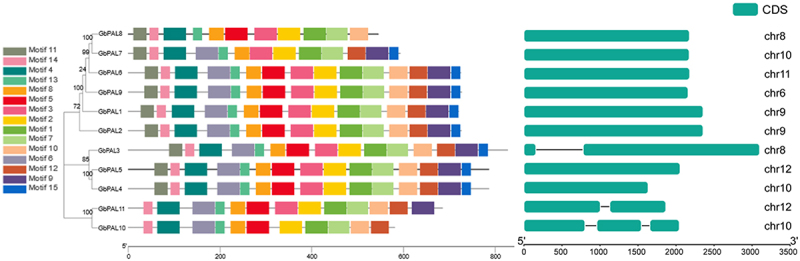
Phylogenetic tree, conservative protein motifs and gene structure of *GbPAL* genes. (a) a phylogenetic tree was constructed for 11 *GbPAL* genes. (b) conservative protein motifs of the 11 *GbPAL* genes. Different conserved motifs with numbers 1–15 were displayed using different colors. (c) gene structure of the 11 *GbPAL* genes.

To investigate the structural diversity of ginkgo *PAL* proteins, we analyzed motif composition and organization using the MEME software (Figure 3). Based on a phylogenetic tree, the 11 *PAL* genes were classified into three subfamilies, and we identified 15 conserved motifs through software analysis, which we named as motif 1–15. In subfamily I, *GbPAL8* lacks motifs 6, 9, 12, and 15, while *GbPAL7* lacks motifs 5 and 10. The motifs in subfamily II are highly conserved, while in subfamily III, *GbPAL11* contains unique motifs 3 and 9 compared to *GbPAL10*. Regarding gene distribution on chromosomes, two genes were located on chromosomes 8, 9, and 12, three genes on chromosome 10, and one gene on chromosomes 6 and 11. Interestingly, 72.7% (8/11) of PAL genes lacked introns.

Taken together, our results suggest that *PAL* genes in ginkgo are concentrated on several chromosomes and belong to the same family, with high conservation among members of different subfamilies. Additionally, our findings imply that different subfamilies may have diverged in function during evolution.

### 3.4. Promoter region of cis-acting elements for GbPAL genes

**Figure 4.**
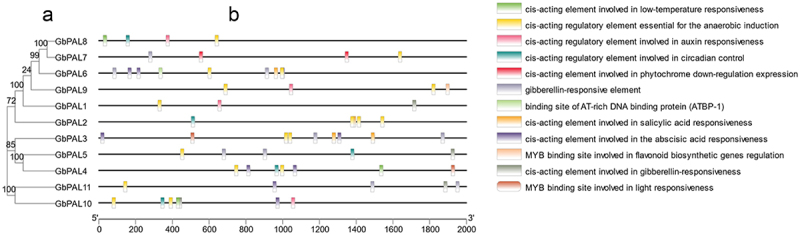
(a) genetic tree of the 11 *GbPAL* genes. (b) cis-regulatory elements are contained in the 11 *GbPAL* genes, with different elements indicated using different colors.

Plant genes are regulated at the transcriptional level through the interaction between cis-acting elements and trans-acting factors, ensuring precise and efficient gene expression. To investigate the potential biological processes involving *GbPAL* genes, we analyzed the 2 kb promoter sequence upstream of 11 *GbPAL* genes using the PlantCARE website (Figure 4). Our analysis revealed a total of 62 cis-acting elements within the promoter region of the *GbPAL* gene family, which can be categorized into four groups: abiotic stress-responsive, hormone-responsive, light-responsive, and specific regulatory elements.

All *GbPALs* were found to contain cis-regulatory elements essential for the induction of anaerobiosis, while low-temperature responsiveness elements were present in *GbPAL11*, *GbPAL8*, and *GbPAL10*. Furthermore, we identified five hormone-related response elements, including those involved in auxin, gibberellin, salicylic acid, and abscisic acid responsiveness. Additionally, MYB binding sites were found to be involved in light responsiveness in *GbPAL4* and *GbPAL3*. Finally, specific regulatory elements were identified, including those involved in circadian control, phytochrome down-regulation expression, and abscisic acid responsiveness. Taken together, these results suggest that *GbPAL* genes may play a crucial role in regulating plant growth, development, and response to abiotic stresses.

### 3.5. Collinearity and protein-protein interaction networks analysis

**Figure 5.**
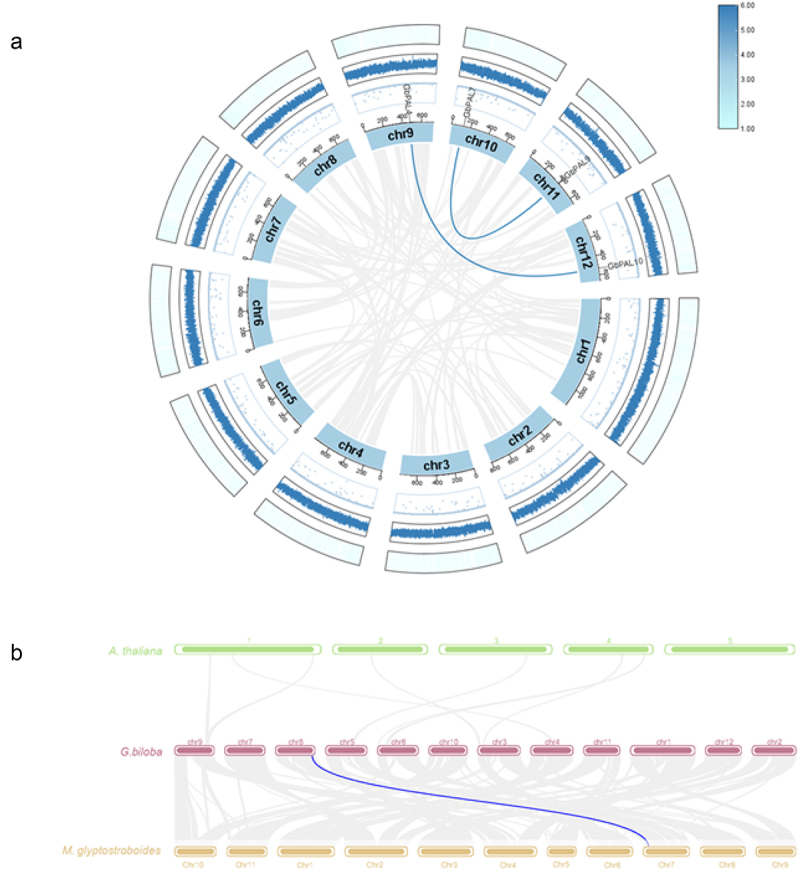
(a) synteny analysis of *GbPAL* genes. The chromosomes of ginkgo are drawn as a circle. The distribution of *GbPAL* genes *is* marked beside the circle. Colored curves indicate the details of syntenic regions between *GbPAL* genes. The circos graph shows chromosomes, gap, GC ratio, and gene density in order from the inside to the outside. (b) Interspecific covariance among members of the *G. biolba*, *A. thaliana* and *M. glyptostroboides*. the blue line indicate the covariance of the *PAL* gene between species.

To further study gene duplication events in the *GbPAL* gene family, covariance analysis was performed. *GbPAL* genes were mainly distributed on chromosomes 6, 8, 9, 10, 11, and 12 of ginkgo, with three genes on chromosome 10 being the most abundant. Analysis of covariance showed the replicative relationships between genes, and relationships within species are shown in Figure 5(a). Two pairs of tandem duplication genes *GbPAL4* (evm.model.chr9.1603)/*GbPAL10* (evm.model.chr12.1405) and *GbPAL7* (evm.model.chr10.571)/*GbPAL9* (evm.model.chr11.1054) were identified.

To determine the covariance of *GbPAL* family individual genes among other species, *A. thaliana* and *Metasequoia glyptostroboides*^27^ were analyzed with the *PAL* family in ginkgo. (Figure 5(b)). Overall, ginkgo has less genome-wide covariance with *A. thaliana* and has no covariant *PAL* genes among them; ginkgo has one covariant *PAL* gene with the same gymnosperm as *M. glyptostroboides*. Comparative analyses of the *GbPAL* family with other species may provide research references for analyzing the genetic relationships and gene functions of the species.

### 3.6. Analysis of GbPAL family transcripts

**Figure 6.**
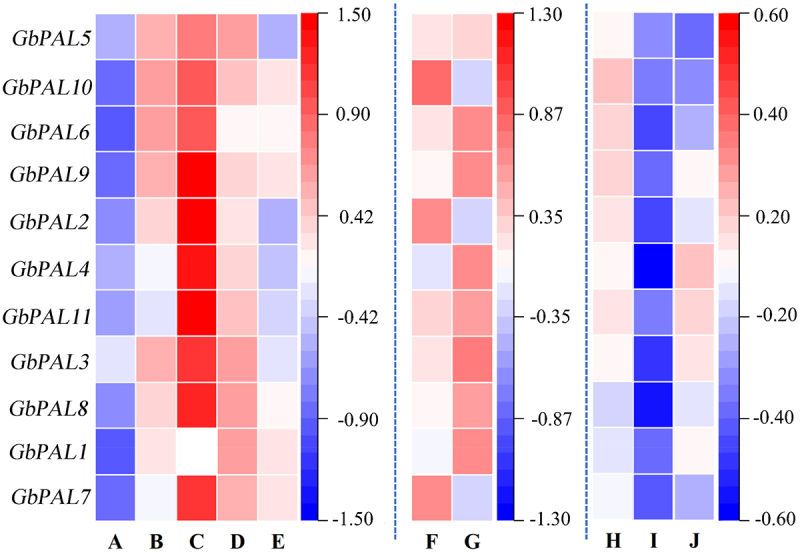
Heatmap of expression of *GbPals* in different states. A: root; B: stem; C: leaf; D: flower; E: seed; F: haploid; G: diploid. H-J: ginkgo leaves under drought stress, salt stress, and high temperature stress.

Gene transcription and expression are important for exploring gene function. According to the heat map of gene expression of *GbPAL* genes in different tissues of ginkgo, it can be seen that *GbPAL* gene have the highest expression level in ginkgo leaves (Figure 6). Analysis of transcriptome data based on haploid versus diploid in the gene expression heat map illustrated that *GbPAL*2, *GbPAL3* and *GbPAL7* expression was upregulated, and the rest of the gene expression was downregulated relative to the diploid. This is consistent with the regular distribution of ginkgo flavonoids in different parts and the ploidy of ginkgo.^23^ Therefore, *GbPAL* genes play an important role in regulating flavonoid accumulation in ginkgo.^28^

Under salt stress, the *GbPALs* exhibited a significant downregulation in expression. Under drought stress, all *GbPALs* except *GbPAL9* and *GbPAL4* were significantly downregulated, while *GbPAL9* and *GbPAL4* showed slight upregulation. Under heat stress, *GbPAL6*, *GbPAL7*, and *GbPAL8* were upregulated, while the others were downregulated. These results suggest that *GbPALs* have different response mechanisms to different stress environments.

### 3.7. qRT-PCR validation of specific expression of GbPAL in different tissues

**Figure 7.**
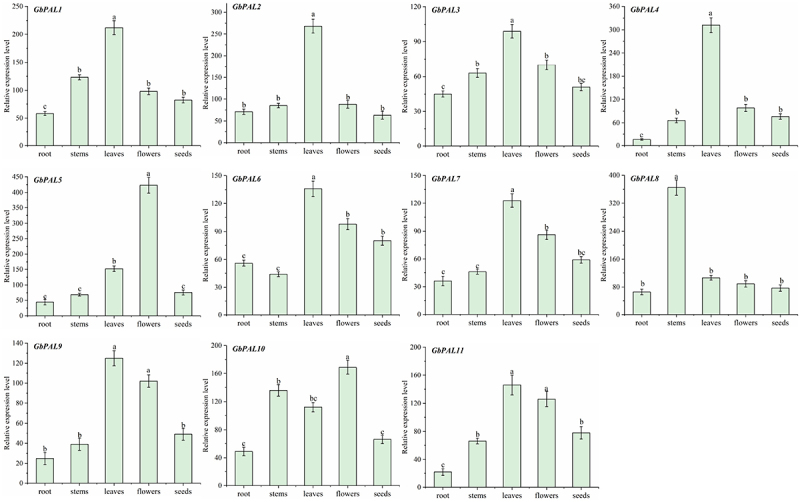
Relative expression levels of *GbPAL* family genes. The vertical bar in the bar graph is standard error. Bars with different letters (a–c) indicate significant differences at *p* < 0.05 according to Duncan’s test. Gene expression values were normalized against the willow *GbCHS-like* gene.

To verify the subspecific expression of the *GbPAL* gene family in different parts of ginkgo, we performed qRT-PCR on 11 *GbPAL* genes (Figure 7). *GbCHS*, as a reference gene stably expressed in different developmental tissues of ginkgo, expressed stably in this experiment. Overall, *GbPAL* was most highly expressed in the leaves, followed by the flowers. For example, in *GbPAL2*, leaf expression is 5.3-fold higher than that of roots and 8-fold higher than that of the seeds. In addition, the expression of *GbPAL5* and *GbPAL10* was the highest in flowers.

**Figure 8.**
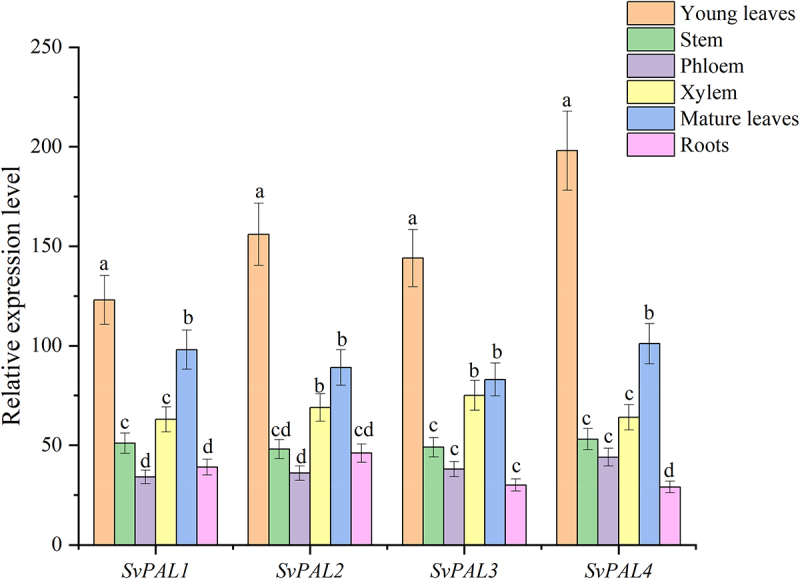
qRT-PCR analysis of the level of expression of *SvPAL1*, *SvPAL2*, *SvPAL3*, and *SvPAL4* in *Salix babylonica* young leaves, stem, phloem, xylem, mature leaves, and root tissue. Gene expression values were normalized against the *SvTIP4* gene.

In order to further compare the differential expression of *PAL* gene family in different parts of plants, we compared the relative expression levels of *PAL* gene family in the leaves, stems, phloem, xylem, mature leaves, and roots of *Salix babylonica* (Figure 8). The results showed that the expression pattern of *GbPALs* in ginkgo was basically consistent. The expression level of *SvPALs* in the leaves was significantly higher than in other parts. The expression level of *SvPALs* decreased with the increasing maturity of leaves.

### 3.8. Protein-protein interactions network analysis of the GbPAL family member

**Figure 9.**
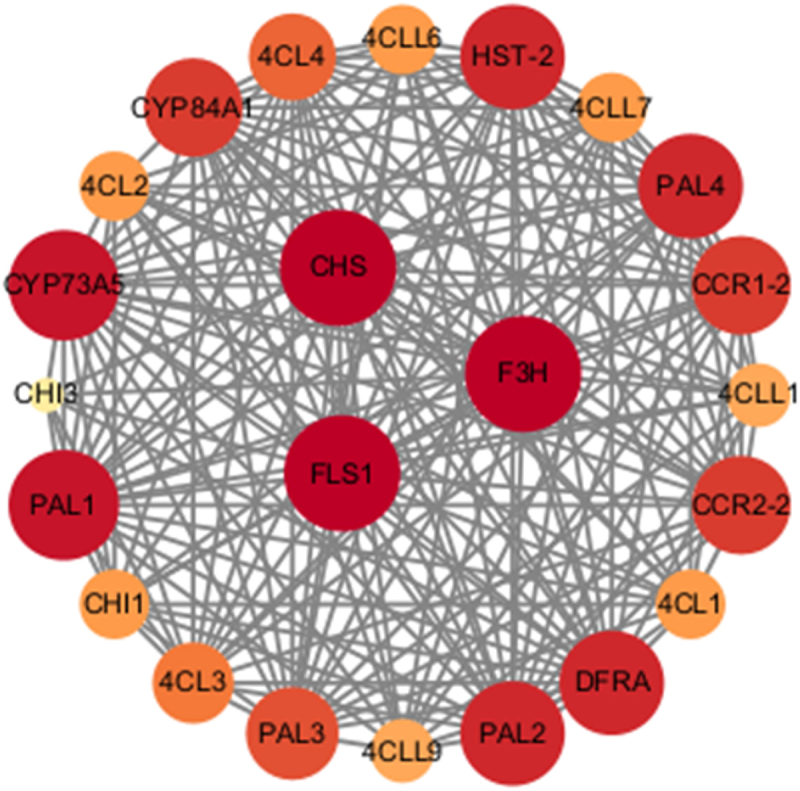
Protein-protein interactions network for GbPAL based on their orthologs in *A. thaliana*.

PAL proteins interactions with CHS, FLS1, and F3H proteins are hightest of the network. CHS is the key enzyme in the biosynthesis pathway of plant flavonoids while F3H acts on the bifurcation of anthocyanins and flavonols.The function of F3H is to converse (2S)-flavanones to (2 R,3 R)-dihydroflavonols that directly intermediate the biosynthesis of flavonols,^29^ FLS1 is involved in the flavonoid synthesis pathway. Networks further indicates that the PAL gene plays an important role in flavonoid synthesis. Previous studies have also shown that CHS, FLS1, F3H, and PAL genes play important roles in stress resistance (Figure 9).^30^

## 4. Discussion

In recent years, mounting evidence has highlighted the crucial role of *PAL* genes in plant growth, resistance, and development.^31^ At the molecular level, a more in-depth understanding of the *PAL* gene family could shed light on potential resistance mechanisms and aid in the development of plant species with enhanced resistance. While the function of the *PAL* gene family has been extensively studied in dicots^32^ and monocots^33,34^ through high-throughput sequencing, research on gymnosperm molecular cloning, characterization, and expression of the *PALs* from ginkgo is limited. Thus, in this study, we conducted a genome-wide characterization of the *PAL* gene in ginkgo, followed by a preliminary analysis of its characteristics.

Members of the *PAL* gene family have been identified and extensively studied in many plants. The *PAL* gene family typically comprises 4–15 members, which varies between species, including alfalfa,^11^ walnut,^35^ potato,^6^ and grape.^12^ The small difference in the number of members in the *PAL* gene family indicates that PAL genes did not undergo significant amplification during species differentiation.^36^ In this study, 11 members of the *PAL* gene family were identified from ginkgo using a bioinformatics approach. Ginkgo mainly contains 12 pairs of chromosomes with a genome size of 9.87 Gb.^18^ This suggests that there is no linear relationship between the number of *PAL* gene family members and the species genome size, and it is speculated that some *PAL* genes evolved to extinction or evolved into other genes during the evolution of ginkgo due to the pressure of natural selection between them.

Gene duplication events are the primary drivers of gene family expansion. In this study, we found that *GbPALs* are distributed in clusters, and a similar clustered distribution of *PAL* chromosome localization exists in watermelon.^37^ In ginkgo, three of the 11 *PALs* were tandemly duplicated on chromosome 10, two tandem genes on chromosomes 8, 9, and 12, and the remaining two were individually arranged on chromosomes 6 and 11. The types of gene duplication are whole genome duplication, homologous exchange, fragment exchange, chromosome fragment exchange, and tandem duplication^37^ *GbPAL4/GbPAL10* and *GbPAL7/GbPAL9* are two pairs of tandem duplicated genes, which may have redundant functions in the biosynthesis of secondary metabolites. *PAL* was amplified in ginkgo, but not in poplar, tobacco, etc., each of which had four *PAL* genes. This suggests that the increase in *PAL* genes in ginkgo may facilitate the improvement of adaptive capacity to the external environment.

The clustering results indicated that *GbPALs* were more closely related to *PaPAL* genes, suggesting that the *GbPAL* gene family existed during the gymnosperm period and may be involved in the flavonoid synthesis pathway, which is similar to *PAL* genes in other plants. We also found that the members of the *GbPAL* gene family, except *GbPAL5* and *GbPAL4*, were independent of monocots and dicots, indicating that the *PAL* gene family existed in gymnosperms before the differentiation of monocots and dicots. *GbPAL3*, *GbPAL11*, and *GbPAL10*, clustered in the same subfamily as *PaPAL*, suggesting that the *PAL* gene family in ginkgo and *PaPAL* gene family may have originated from the same ancestor. In addition, *GbPAL1*, *GbPAL2*, *GbPAL6*, *GbPAL7*, *GbPAL8*, and *GbPAL9* genes formed separate subfamilies and were highly conserved throughout evolution.

We found that the *PAL* gene family contains not only a large number of abiotic stress elements but also various types of regulatory elements such as light and phytohormone response elements. Different *PAL* family members contain different numbers and types of elements, indicating that *GbPAL* genes are involved in different biological regulatory processes, and different *PAL* genes have specific regulatory patterns. Many studies have shown that *PAL* genes are involved in responses to environmental stimuli, including stress conditions such as UV exposure,^1^ drought,^38^ waterlogging,^11^ pathogens, wounds, and extreme temperatures. Individual PAL proteins may be structurally distinct from other PALs and appear to have different functions, making *PAL* a multifunctional gene family. For example, in *Arabidopsis*, *AtPAL1–4* are mainly involved in lignin synthesis, whereas *AtPAL1* and *AtPAL2* are associated with flavonoid synthesis.^39^

In our previous study, it was found that the flavonoid content of haploid leaves was always smaller than that of diploids, and transcriptomic and proteomic correlation analyses yielded that the PAL gene interacts with several other genes and plays a major role in haploid ginkgo flavonoid regulation. Therefore, we again analyzed the expression of each member of the *PAL* gene family specifically through transcriptomic data, and the expression of *GbPAL2*, *GbPAL7* and *GbPAL10* was upregulated, while the expression of the remaining genes was downregulated relative to the diploid.

As the initial enzyme in the phenylpropanoid pathway, *PAL* is tightly regulated both pre- and post-transcriptionally.^35^ Through protein interaction network prediction, we found that the protein encoded by PAL gene interacts with proteins such as CHS, F3H, FLS3 in the phenylpropanoid metabolism pathway, further indicating that PAL plays a crucial role in flavonoid synthesis. Expression of *PAL* genes in various tissues has been extensively studied in different plant species. In our study, we found that *GbPAL* was constitutively expressed in all tissues examined, and our transcriptome data were in agreement with the qRT-PCR results. Generally, *GbPAL* expression was highest in leaves (excluding *GbPAL4* and *GbPAL10*) and lowest in roots and seeds, which is consistent with previous reports.^40^ However, it has also been reported that in Jatropha curcas, *PAL1* is highly expressed in flowers, indicating a potential role in secondary metabolic activity.^41^ Thus, it is speculated that *GbPAL4* and *GbPAL10* may play a relatively significant role in secondary metabolic activity during flowering.^42^

## 5. Conclusions

In this study, we investigated the *PAL* gene family in ginkgo at the genome-wide level using bioinformatics tools. The genome of ginkgo displayed amplification of *PAL* genes, with 11 genes identified and unevenly distributed across the genome. Additionally, *GbPALs* were classified into three subfamilies, which are evolutionarily conserved. Members belonging to the same subfamily exhibited similar biosynthetic structures and conserved protein motifs. The analysis of cis-elements indicated that *GbPALs* may play a crucial role in plant growth and development, particularly in response to hormones, light, and abiotic stresses. Thus, our findings provide novel insights into the *GbPAL* gene family and pave the way for further investigation of its biological functions.

## Supplementary Material

Supplemental Material
