# Supplementary figures and images for: Expression profiling of the phenylalanine ammonia-lyase (PAL) gene family in ginkgo biloba L
Source: Plant Signal Behav. 2023 Oct 30;18(1):2271807. doi: 10.1080/15592324.2023.2271807 (PMC10761125; doi:10.1080/15592324.2023.2271807)

## Slide 1
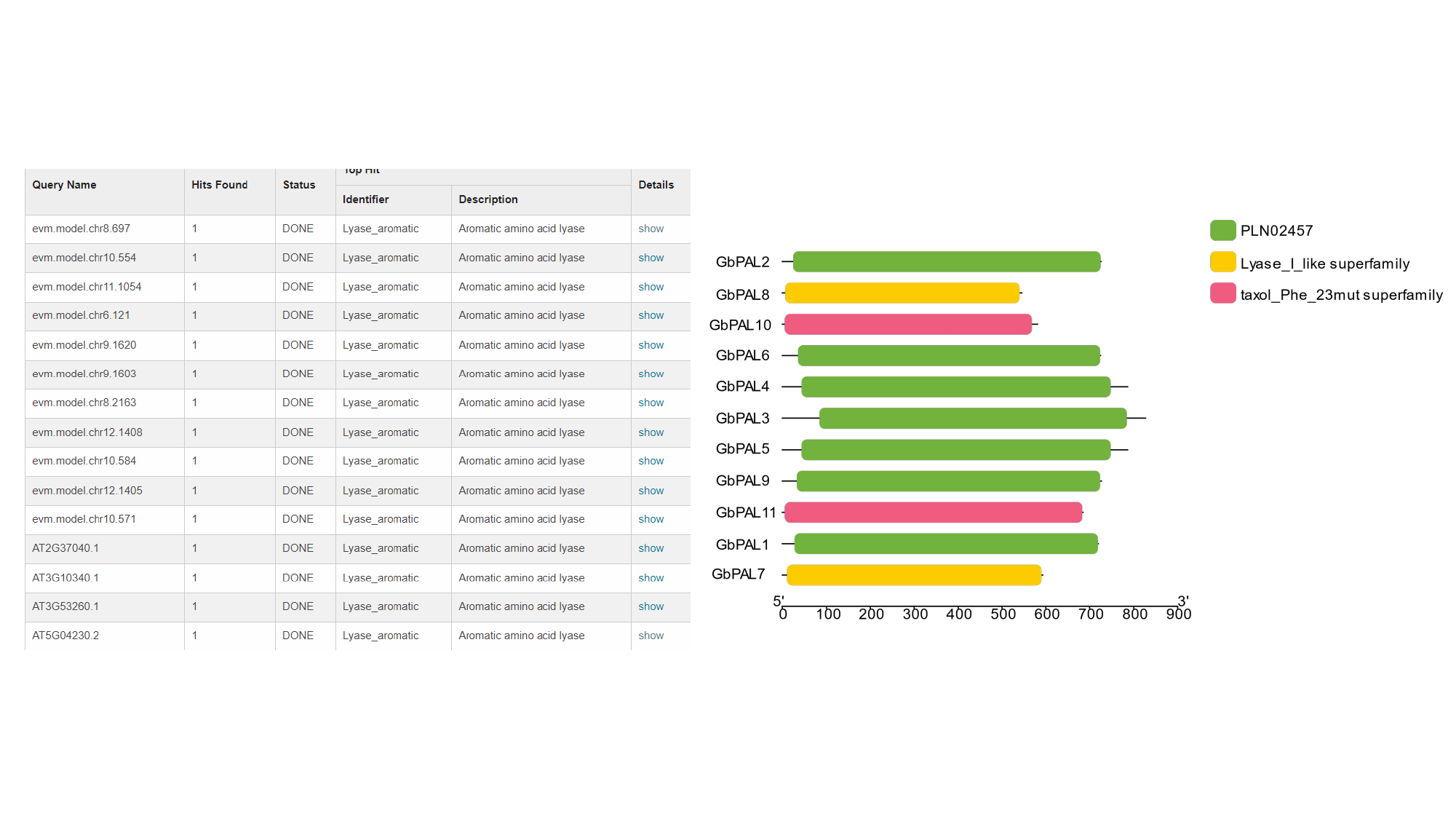

Supplement: Supplemental Material [file KPSB_A_2271807_SM1680.zip › Supplementary/Supplementary Figure. 1.pptx]
